# Supplementary material for: Point mutations in SARS-CoV-2 variants induce long-range dynamical perturbations in neutralizing antibodies
Source: Chem Sci. 2022 May 23;13(24):7224–39. doi: 10.1039/d2sc00534d (PMC9214918; doi:10.1039/d2sc00534d)
Supplement: SC-013-D2SC00534D-s001 [file SC-013-D2SC00534D-s001.pdf]

Electronic Supplementary Material (ESI) for Chemical Science.
This journal is © The Royal Society of Chemistry 2022

Supporting information for "Point Mutations in SARS-CoV-2 Variants Induce Long-Range Dynamical Perturbations in Neutralizing Antibodies"

Dhiman Ray,[*,†] Riley Nicolas Quijano,[†] and Ioan Andricioaei[*,†,‡]

†*Department of Chemistry, University of California Irvine, Irvine CA 92697*

‡*Department of Physics and Astronomy, University of California Irvine, Irvine CA 92697*

E-mail: dray1@uci.edu; andricio@uci.edu

Table 1: A list of simulations performed in the current study

System	Variant	Strain	RBD Mutations	Unbiased[a] MD	Number[a] of Atoms	SMD[b]	Number[b] of Atoms
RBD only	WT	WT	-	0.3μs×1	31392	-	-
	alpha	B.1.1.7	N501Y	0.3μs×1	31477	-	-
	beta	B.1.351	N501Y,K417N,E484K	0.3μs×1	31384	-	-
	kappa	B.1.617	L452R,E484Q	0.3μs×1	31165	-	-
	delta	B.1.617.2	L452R,T478K	0.3μs×1	31716	-	-
RBD	WT	WT	-	0.5μs×1	76753	10ns×5	9513
+	alpha	B.1.1.7	N501Y	0.5μs×1	76916	10ns×5	9520
B38 Ab	beta	B.1.351	N501Y,K417N,E484K	0.5μs×1	77500	10ns×5	9519
	kappa	B.1.617	L452R,E484Q	0.5μs×1	75110	10ns×5	9526
	delta	B.1.617.2	L452R,T478K	0.5μs×1	80889	10ns×5	9532
RBD	WT	WT	-	0.5μs×1	52625	10ns×5	6491
+	alpha	B.1.1.7	N501Y	0.5μs×1	50291	10ns×5	6498
BD23 Ab	beta	B.1.351	N501Y,K417N,E484K	0.5μs×1	53669	10ns×5	6497
	kappa	B.1.617	L452R,E484Q	0.5μs×1	53782	10ns×5	6504
	delta	B.1.617.2	L452R,T478K	0.5μs×1	55535	10ns×5	6510

[a] Performed in explicit solvent molecules and ions.
[b] Performed in implicit solvent environment

Steered Molecular Dynamics in Explicit Solvent

Methods

To establish the validity of our implicit solvent SMD approach, we performed additional steered molecular dynamics simulations in an explicit solvent environment. The solvated structures were prepared following a protocol similar to that used for unbiased MD simulations, except we added 50 Å of water padding in one side along the principal axis of the RBD-Antibody complex, to allow for sufficient solvent coverage for the dissociating system. The pulling speed was kept at 1 Å/ns along the center of mass distance based CV to allow for sufficient time for the relaxation of explicit water molecules. This protocol led to a simulation time pf 40 ns for each complex. As these simulations are significantly more expensive compared to the implicit solvent SMD runs, only one set of pulling simulation was conducted for the WT and the *kappa* variant for each antibody. The choice of the *kappa* variant was motivated by the fact that in implicit solvent environment, this variant showed the highest

change in the non-equilibrium work needed for detachment from the antibody in comparison to the WT.

Results

The mutations in the *kappa* variant reduces the non-equilibrium work required for the dissociation of both the BD23 and B38 antibody (Fig. S26). This observation is consistent with our findings from the implicit solvent SMD simulations. Due to the presence of explicit water and a lower pulling force, we cannot expect the absolute value of the work to be identical. The dissociation mechanism of the RBD-antibody complex obtained from the implicit solvent and explicit solvent SMD simulation are in qualitative agreement with each other. In both cases, the RBD loop consisting of residues 470-490 works like an anchor to hold on to the antibody, immediately prior to the detachment (Fig. S27). The S477 residue of WT RBD and the A475 residue of the *kappa* variant RBD forms the last hydrogen bond with the B38 antibody during the force induced dissociation process, in both explicit and implicit solvent simulations. Although, the E484 of WT RBD form the last H-bond with the departing BD23 antibody under both conditions, the last connecting residue is different for the *kappa* RBD in the explicit (N481) and implicit (S477) environment. Although this does not change our overall conclusion that the mutations in RBD significantly perturbs the dissociation mechanism because the 484 residue which form hydrogen bond with the BD23 antibody looses its capability to form the hydrogen bond when it is mutated.

Supporting Figures

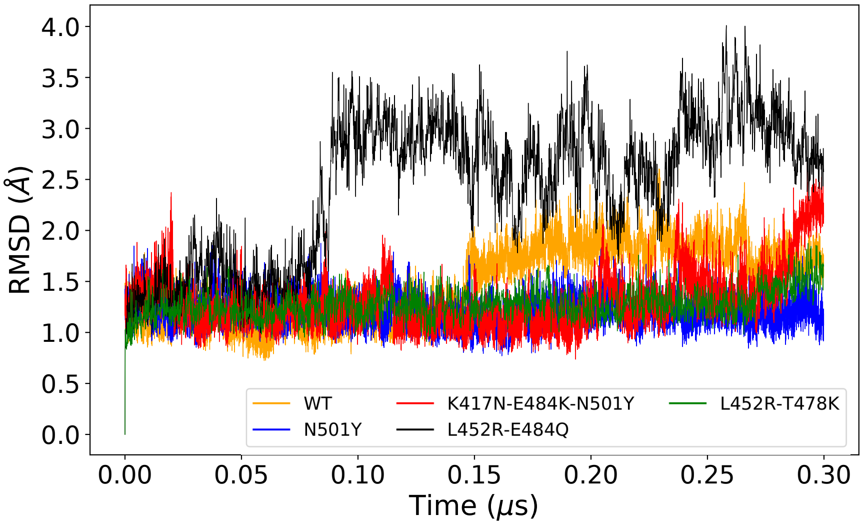

Figure S1: Root mean squared deviations of the bare RBD systems (WT and all mutants) from the long unbiased MD simulation.

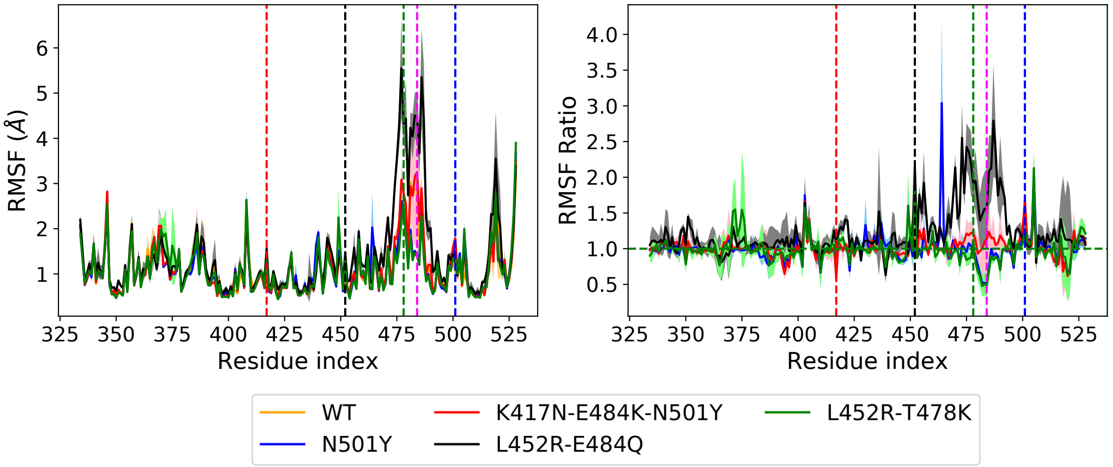

Figure S2: The root mean squared fluctuations (RMSF) of all residues of the bare RBD systems (WT and mutants) and the residue wise RMSF ratio for the three mutant systems.

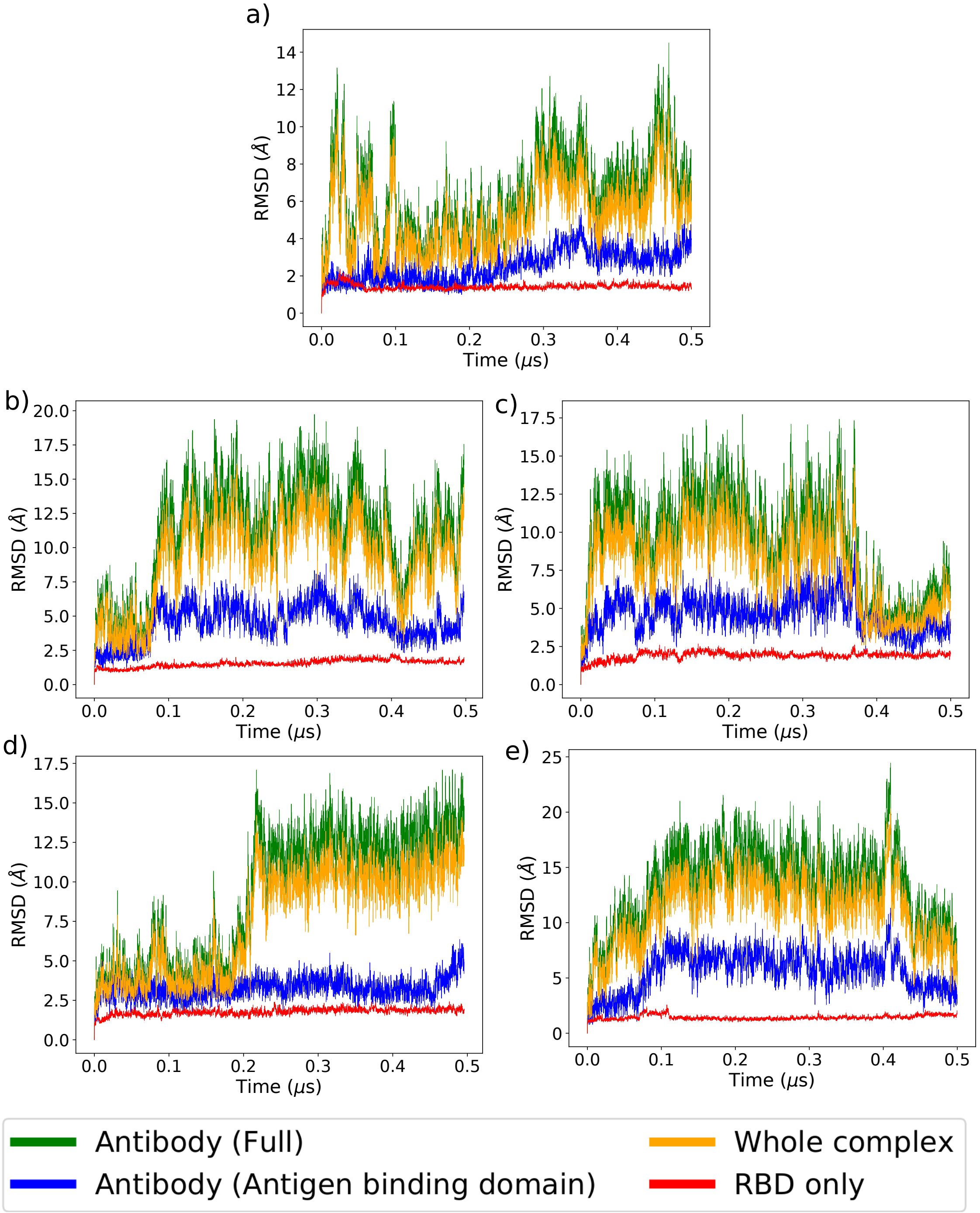

Figure S3: RMSD of different domains of the B38 Fab complex with (a) WT, (b) N501Y mutant, (c) K417N-E484K-N501Y mutant, (d) L452R-E484Q, and (e) L452R-T478K mutant forms of RBD from the long unbiased MD simulation.

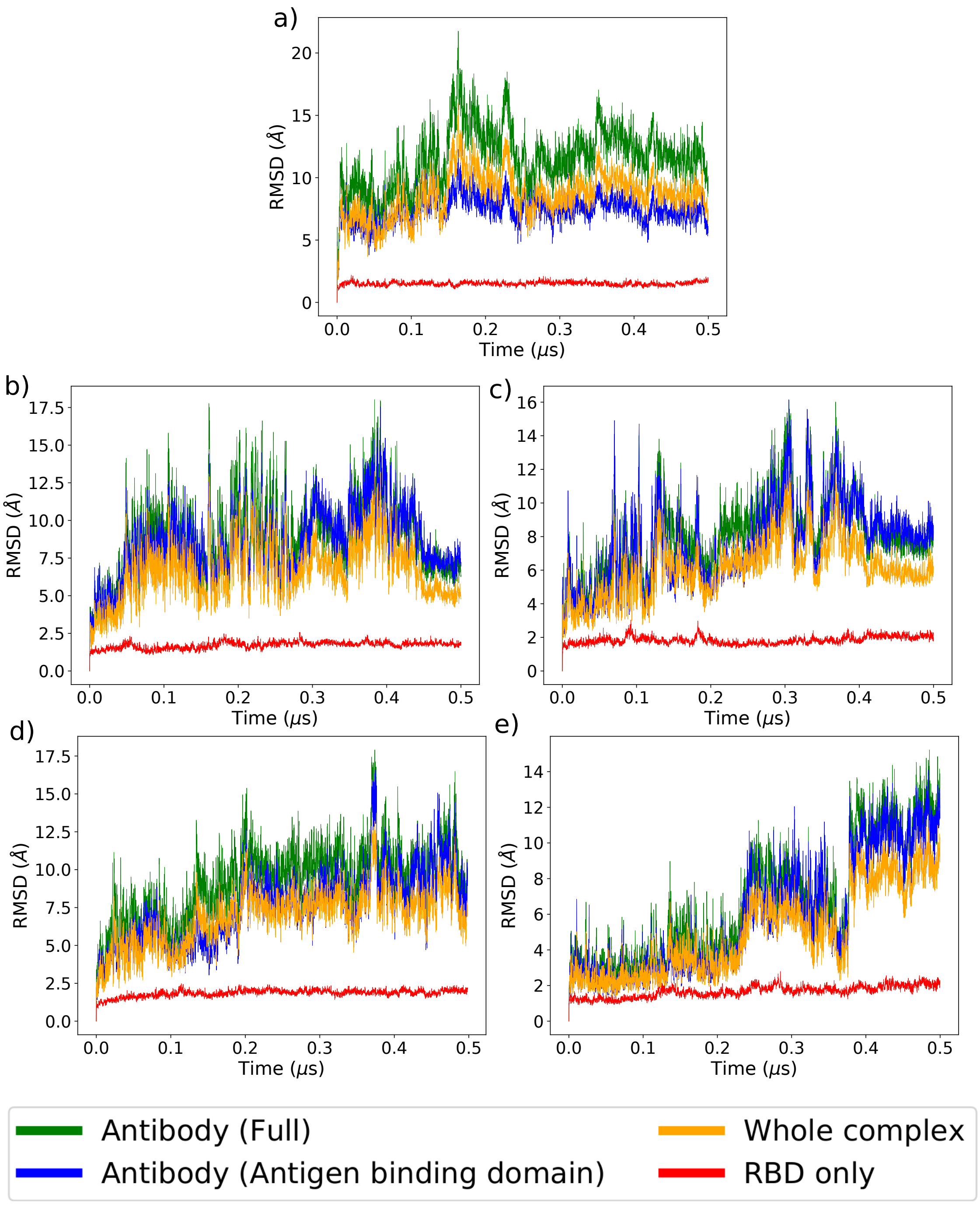

Figure S4: RMSD of different domains of the BD23 Fab complex with (a) WT, (b) N501Y mutant, (c) K417N-E484K-N501Y mutant, (d) L452R-E484Q, and (e) L452R-T478K mutant forms of RBD from the long unbiased MD simulation.

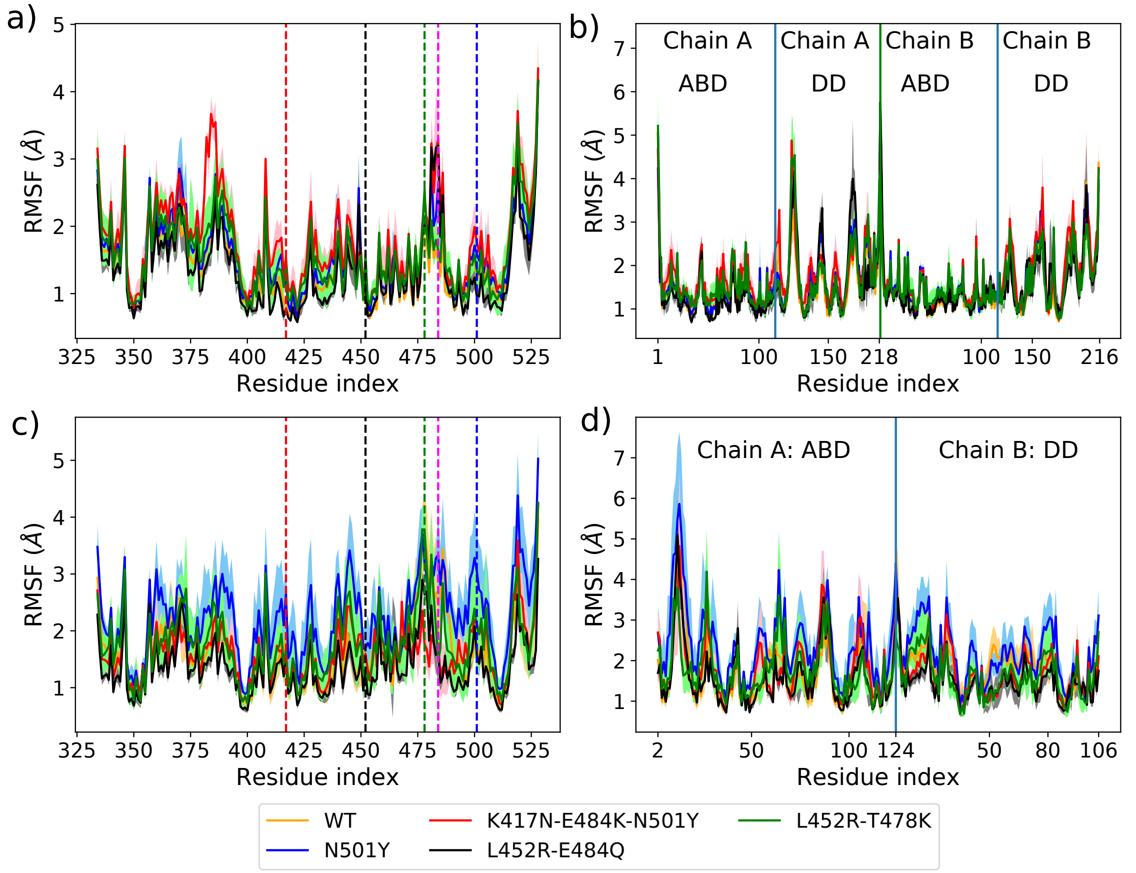

Figure S5: Residue-wise RMSF of all the RBD-antibody complexes: (a) RBD and (b) the antibody part of the RBD-B38 Fab complex and the (c) RBD and (d) the antibody part of the RBD-BD23 Fab complex, for the WT and the mutants. In (a) and (c) the location of mutated residues are marked in dashed line with the following color code; red: 417, black: 452, green: 478, pink: 484 and blue: 501.

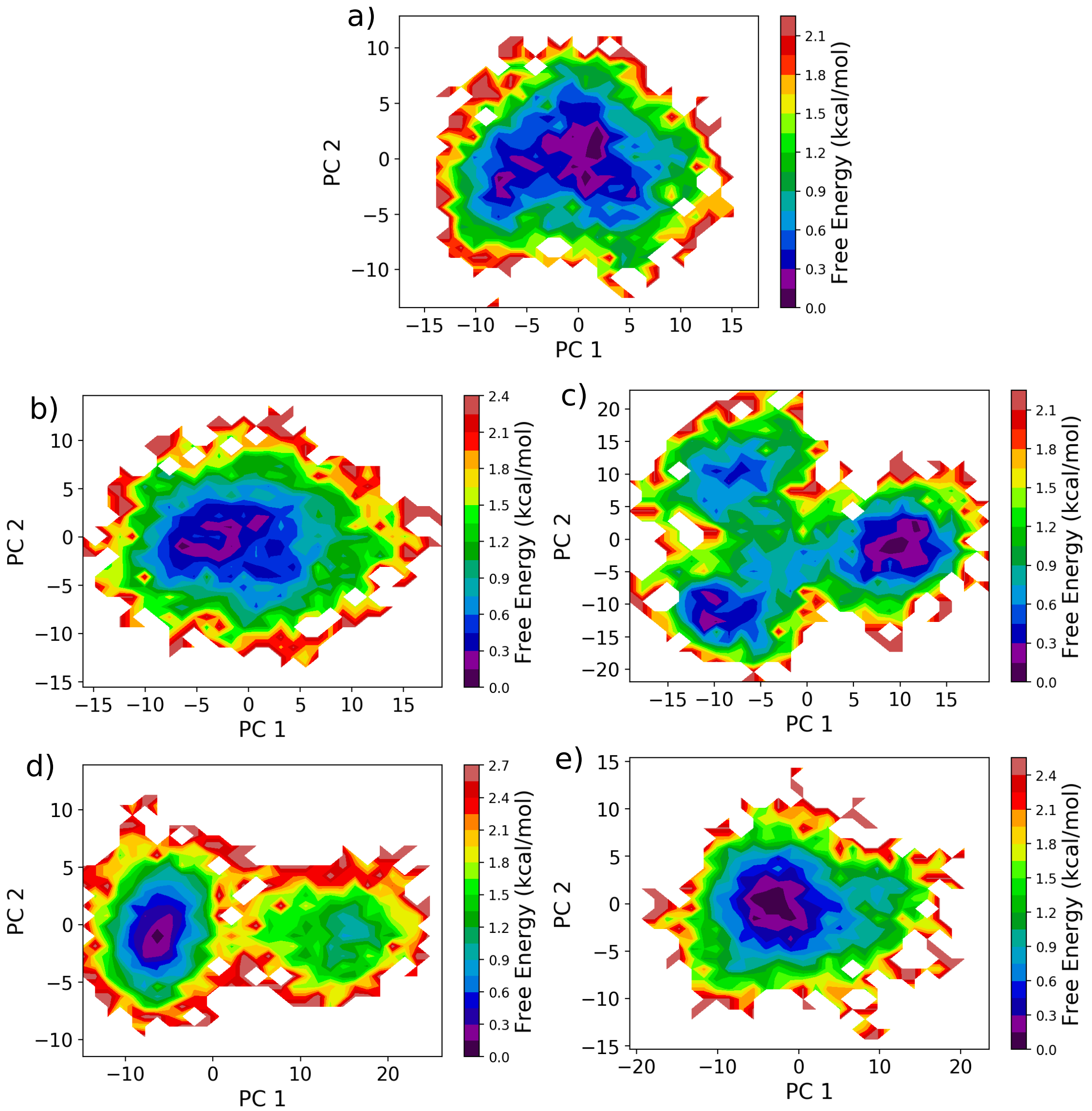

Figure S6: The free energy landscape of the RBD-B38 Fab complexes projected along the first two principal components, for (a) the WT, (b) N501Y mutant, (c)K417N-E484K-N501Y mutant, (d) L452R-E484Q, and (e) L452R-T478K mutant forms.

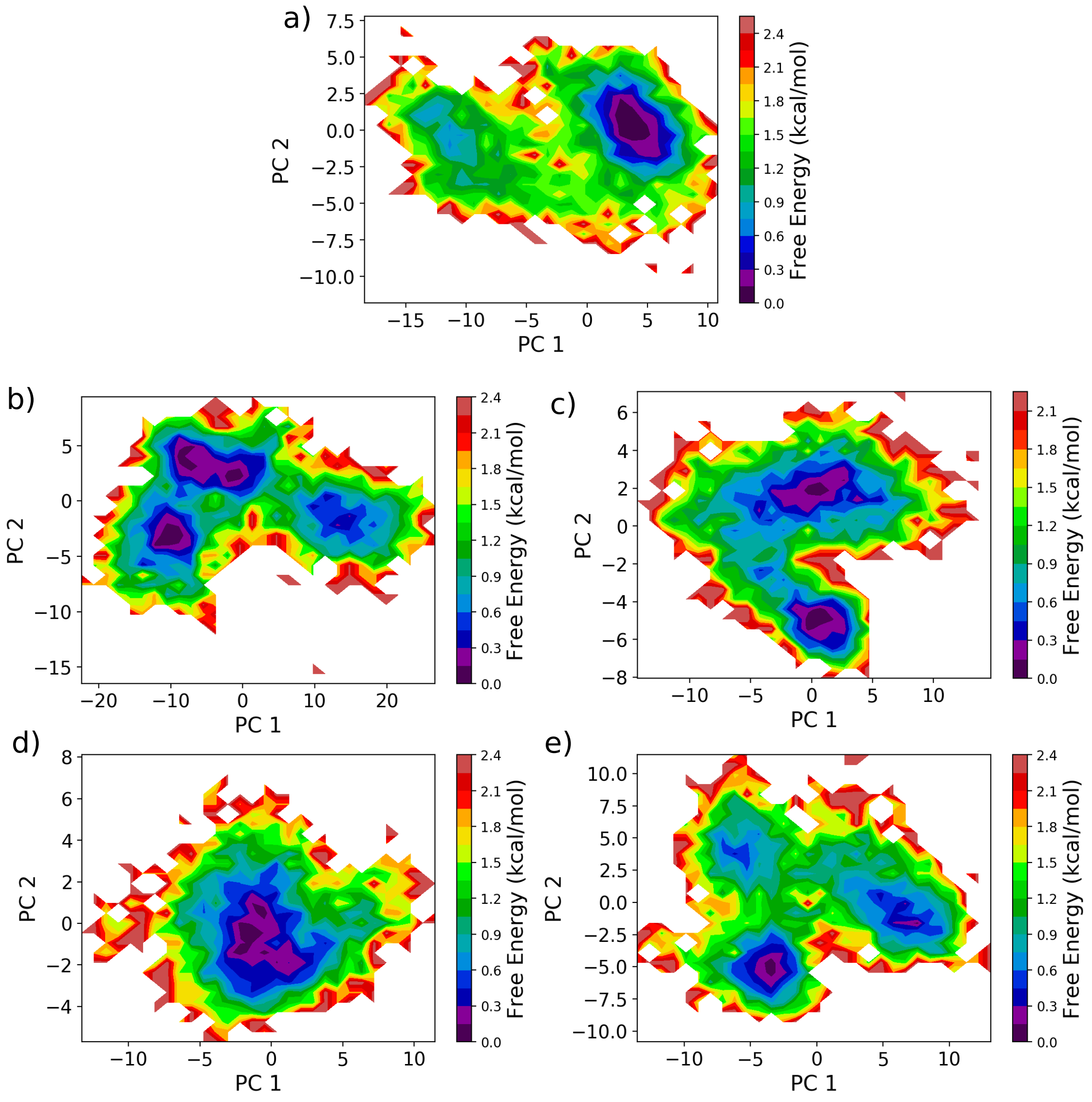

Figure S7: The free energy landscape of the RBD-BD23 Fab complexes projected along the first two principal components, for (a) the WT, (b) N501Y mutant, (c)K417N-E484K-N501Y mutant, (d) L452R-E484Q, and (e) L452R-T478K mutant forms.

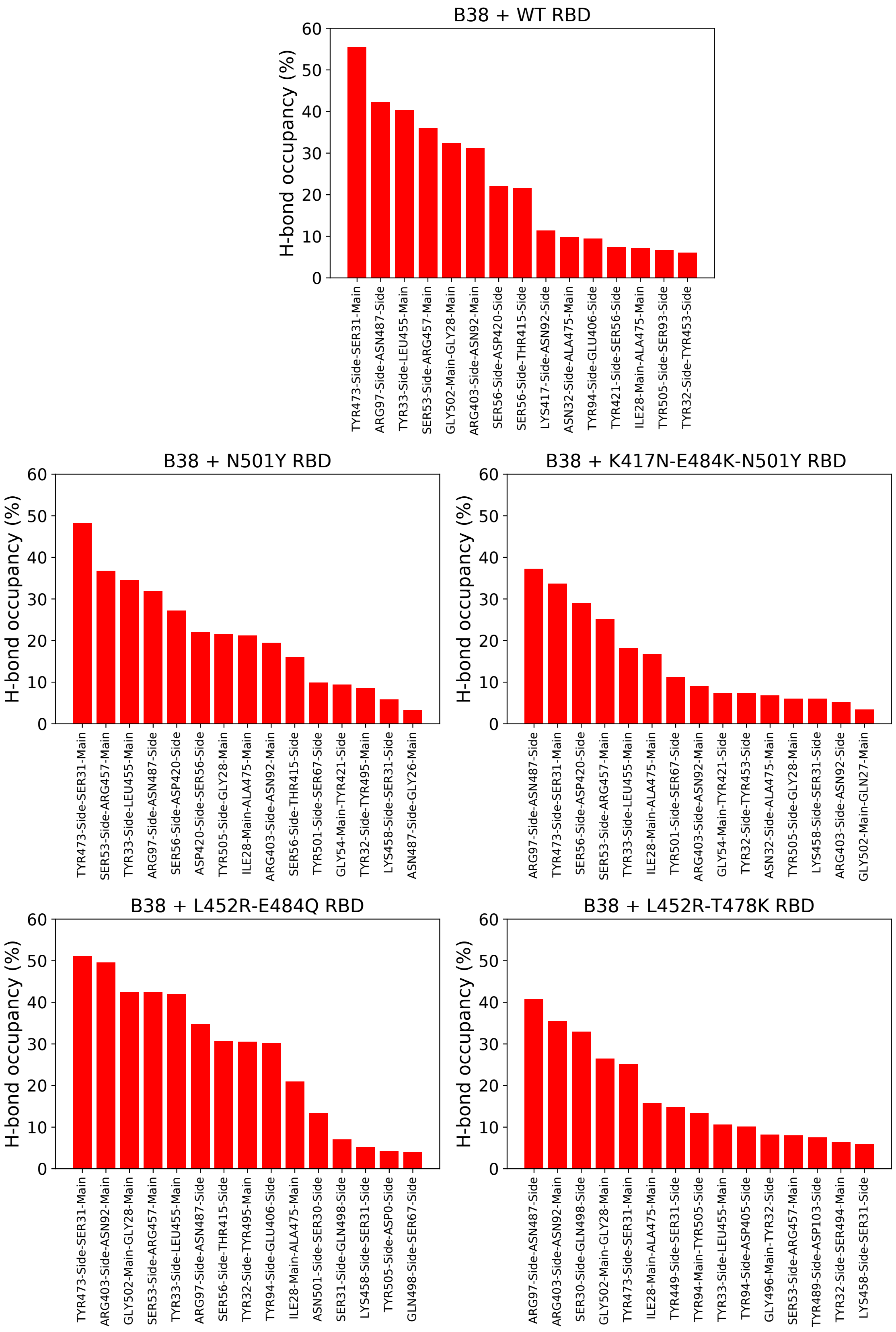

Figure S8: The occupancy of the most prevalent hydrogen bonds between the different variants of the spike RBD and the B38 antibody, sampled from long unbiased simulation.

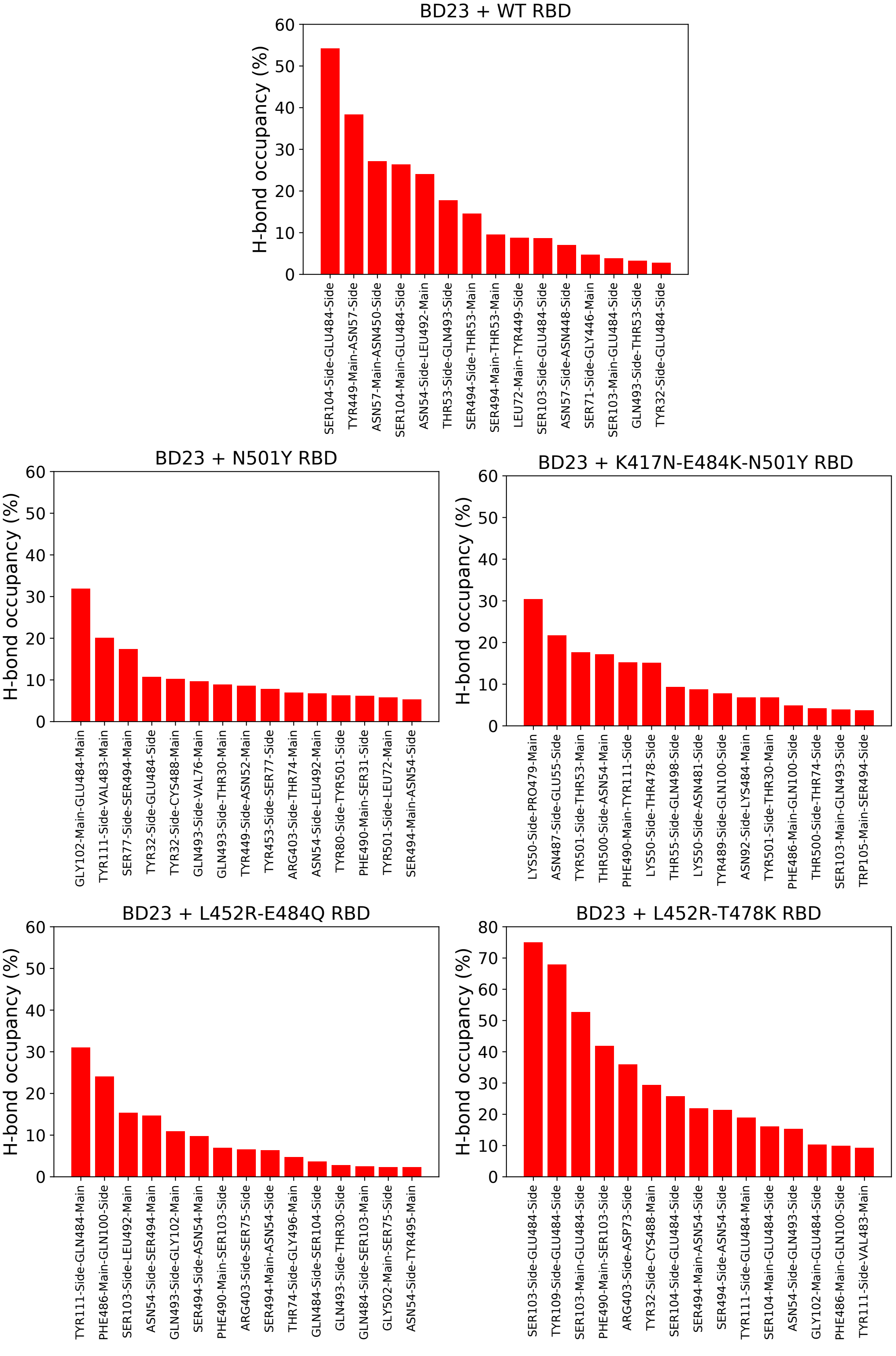

Figure S9: The occupancy of the most prevalent hydrogen bonds between the different variants of the spike RBD and the BD23 antibody, sampled from long unbiased simulation.

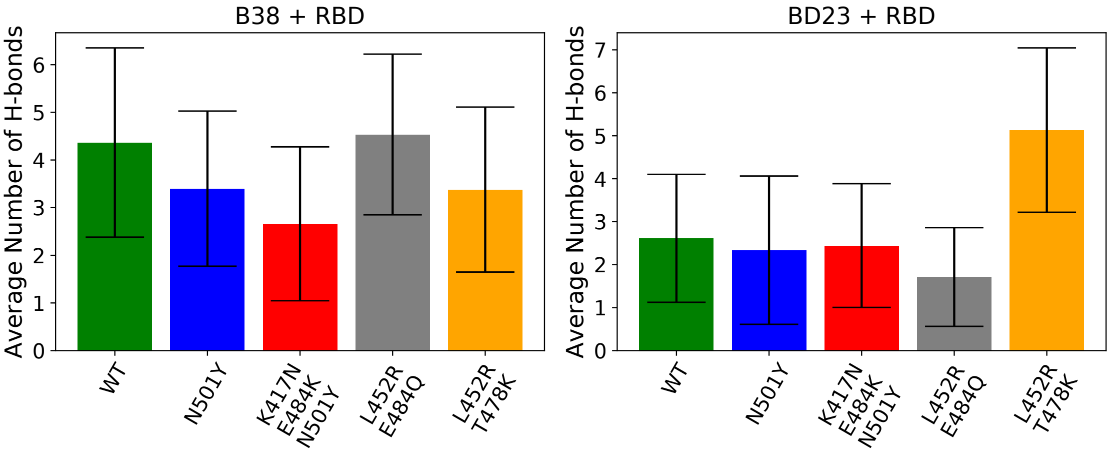

Figure S10: The average number of hydrogen bonds between different variants of the spike RBD and the two antibodies.

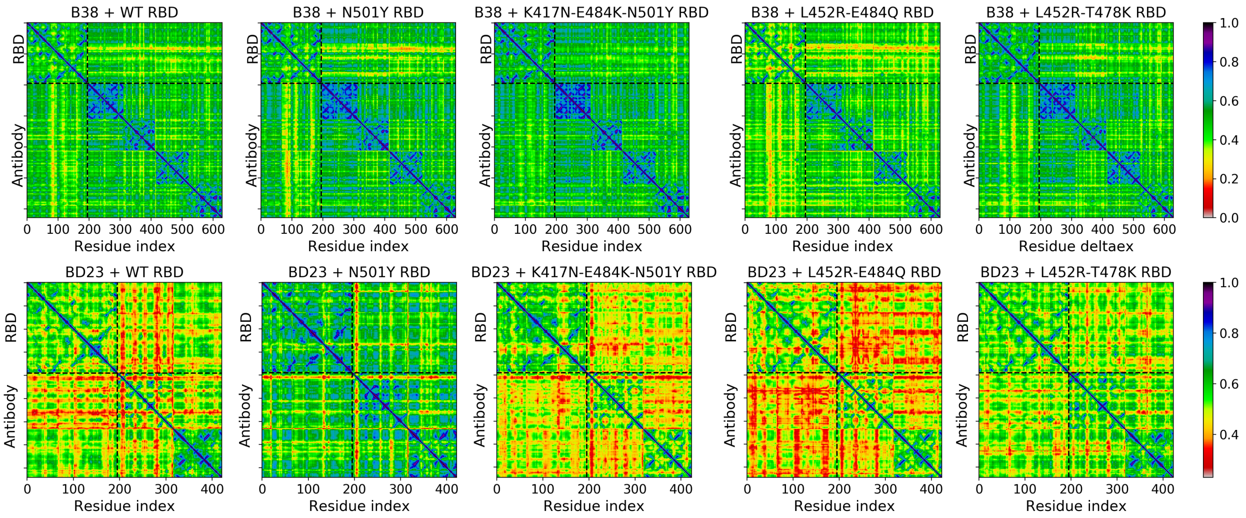

Figure S11: Linear mutual information based cross-correlation between all residue pairs in the RBD-antibody complexes for both the antibodies and all four variants of the RBD.

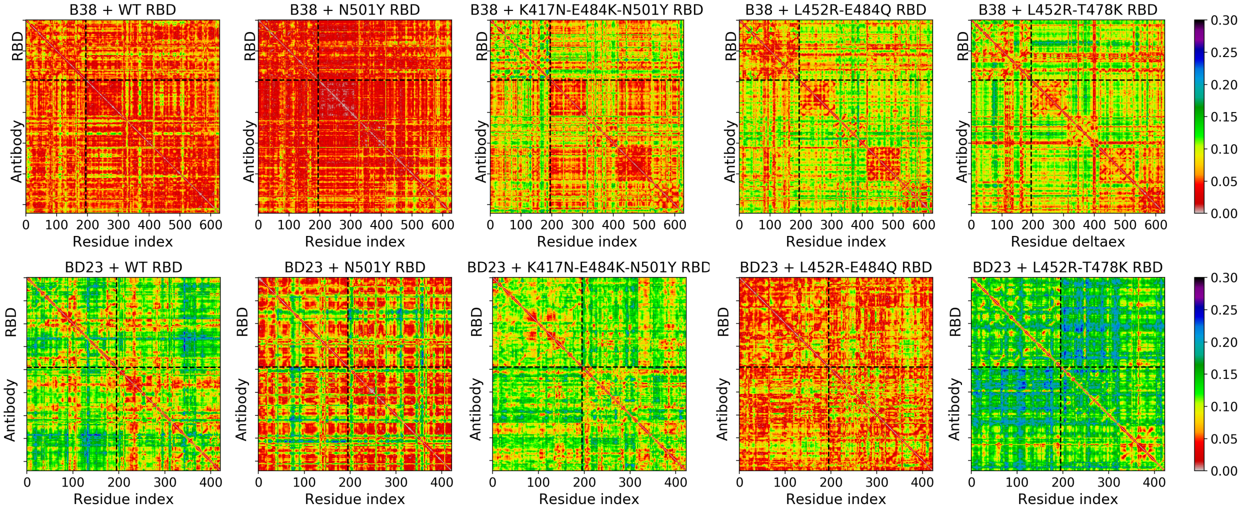

Figure S12: The standard deviation of the linear mutual information based cross-correlation between all residue pairs in the RBD-antibody complexes for both the antibodies and all four variants of the RBD. The standard deviation has been computed over four 100 ns trajectory segments (see Methods section in main manuscript)

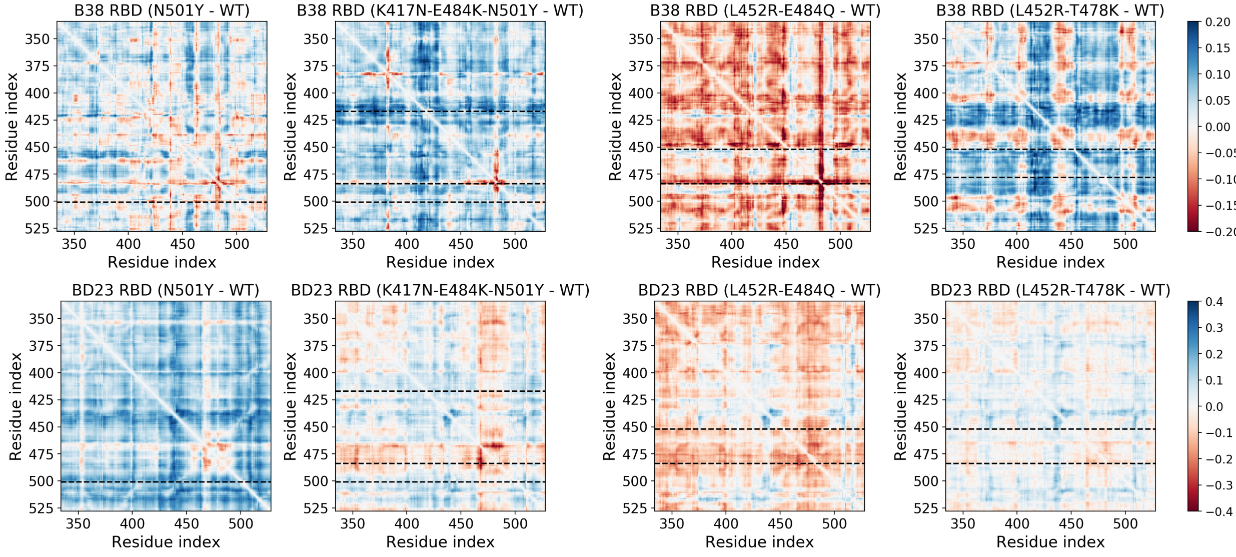

Figure S13: The change in the linear mutual information based cross-correlation between all residue pairs in only the RBD region for different variants and for two antibody systems. The WT-RBD system is considered as reference.

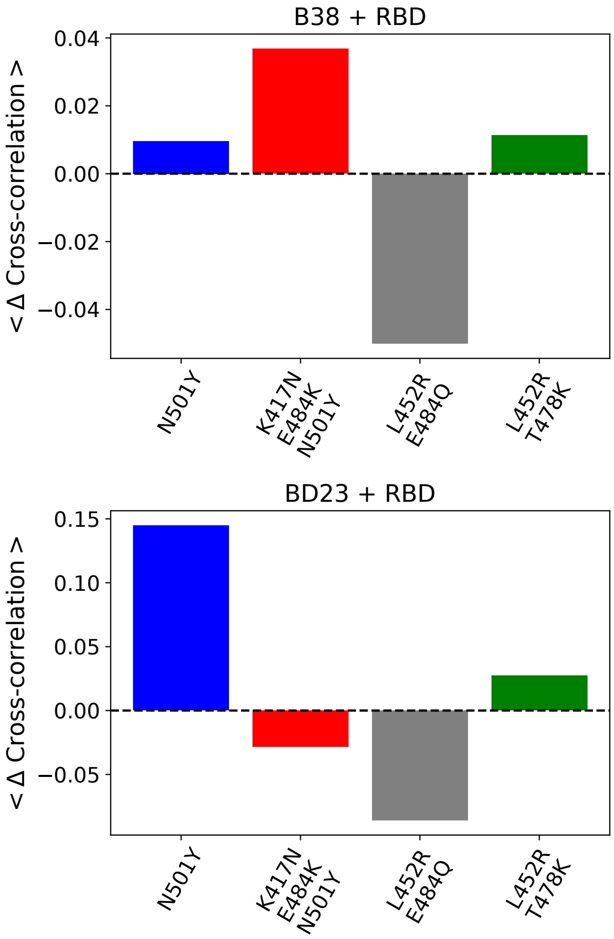

Figure S14: The average change in cross-correlation values per residue for the four mutants in complex with the B38 and BD23 antibody.

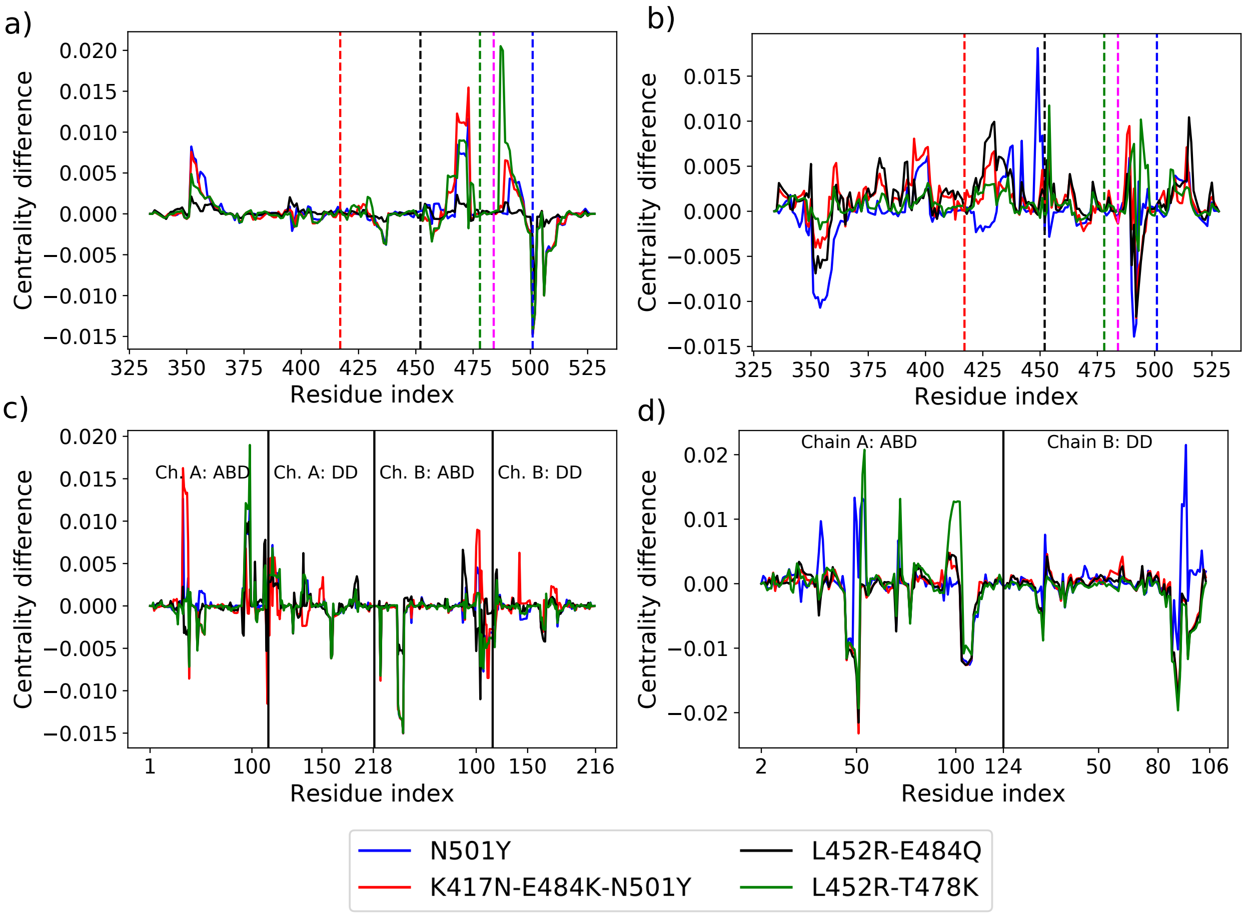

Figure S15: The change in betweenness centrality for all the residues of the mutant RBD complexed with the two antibodies. The upper panel depicts the BC change for only the RBD part for the (a) B38 complex and (b) BD23 complex. The location of the mutations are shown as vertical dashed lines. The lower panel depicts the same for only the antibody part for the (a) B38 complex and (b) BD23 complex.

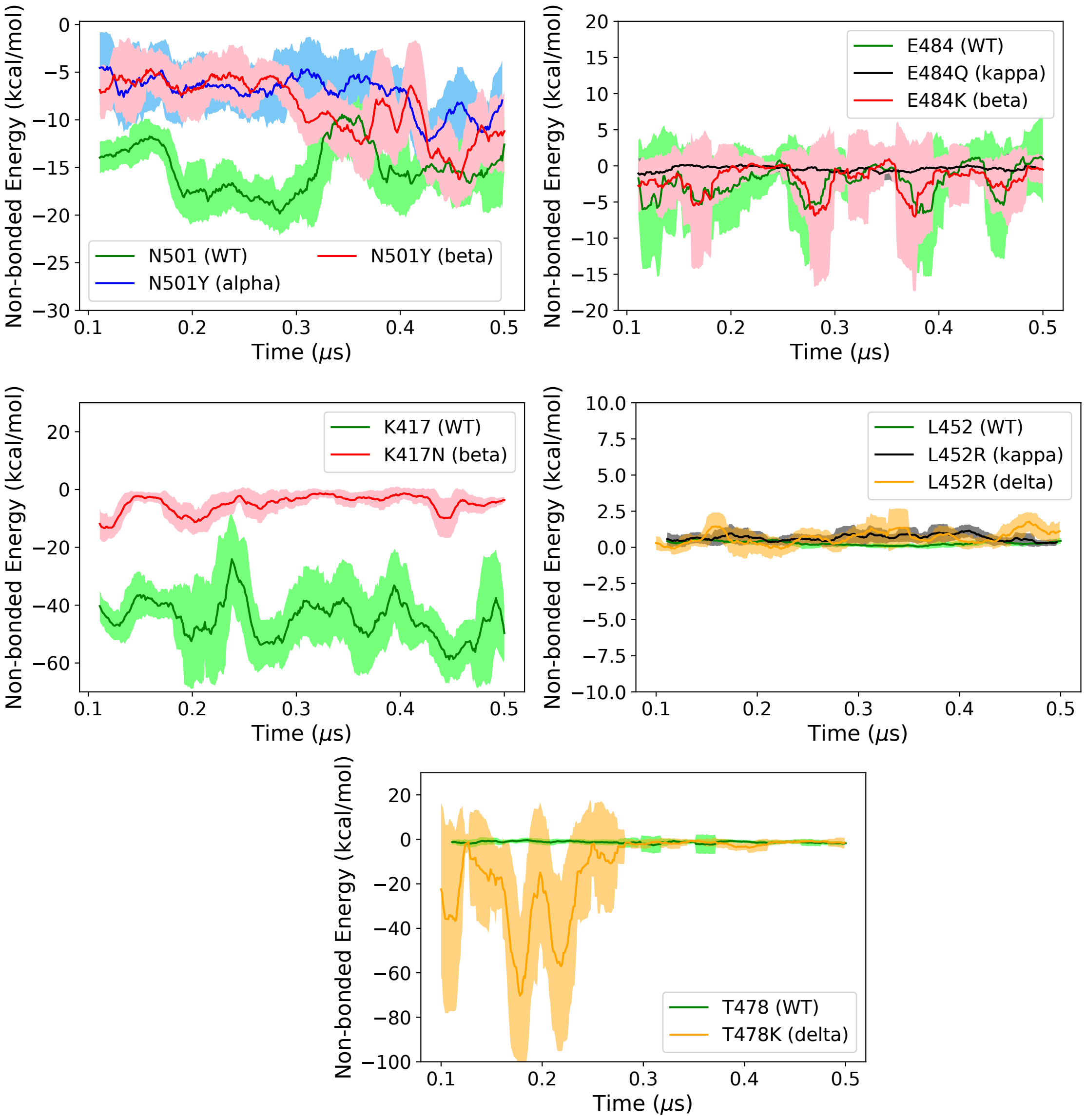

Figure S16: The non-bonded interaction energy between the B38 antibody and each individual RBD residue, mutated in the different variants. The error bars are running average over 20 ns.

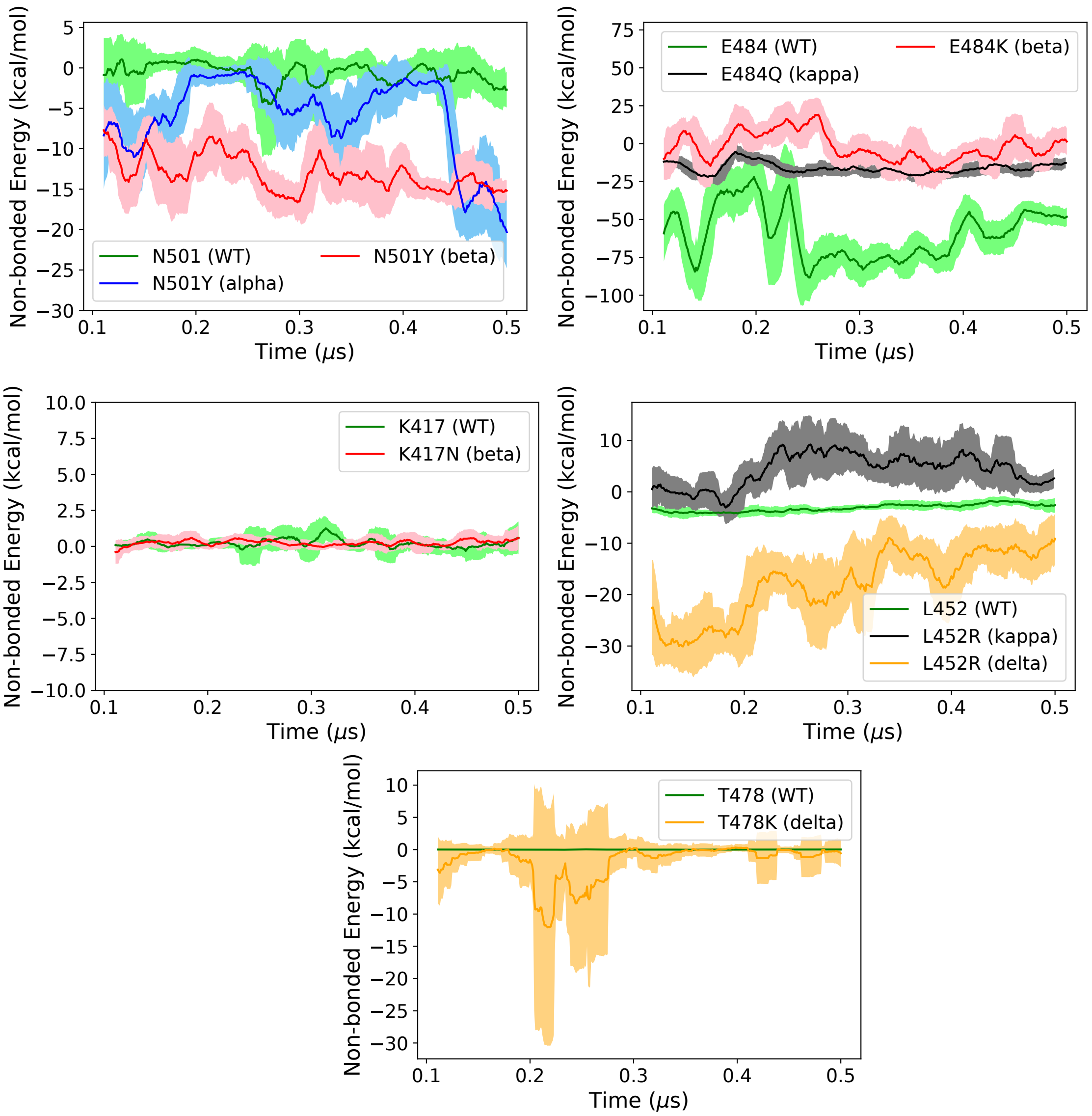

Figure S17: The non-bonded interaction energy between the BD23 antibody and each individual RBD residue, mutated in the different variants. The error bars are running average over 20 ns.

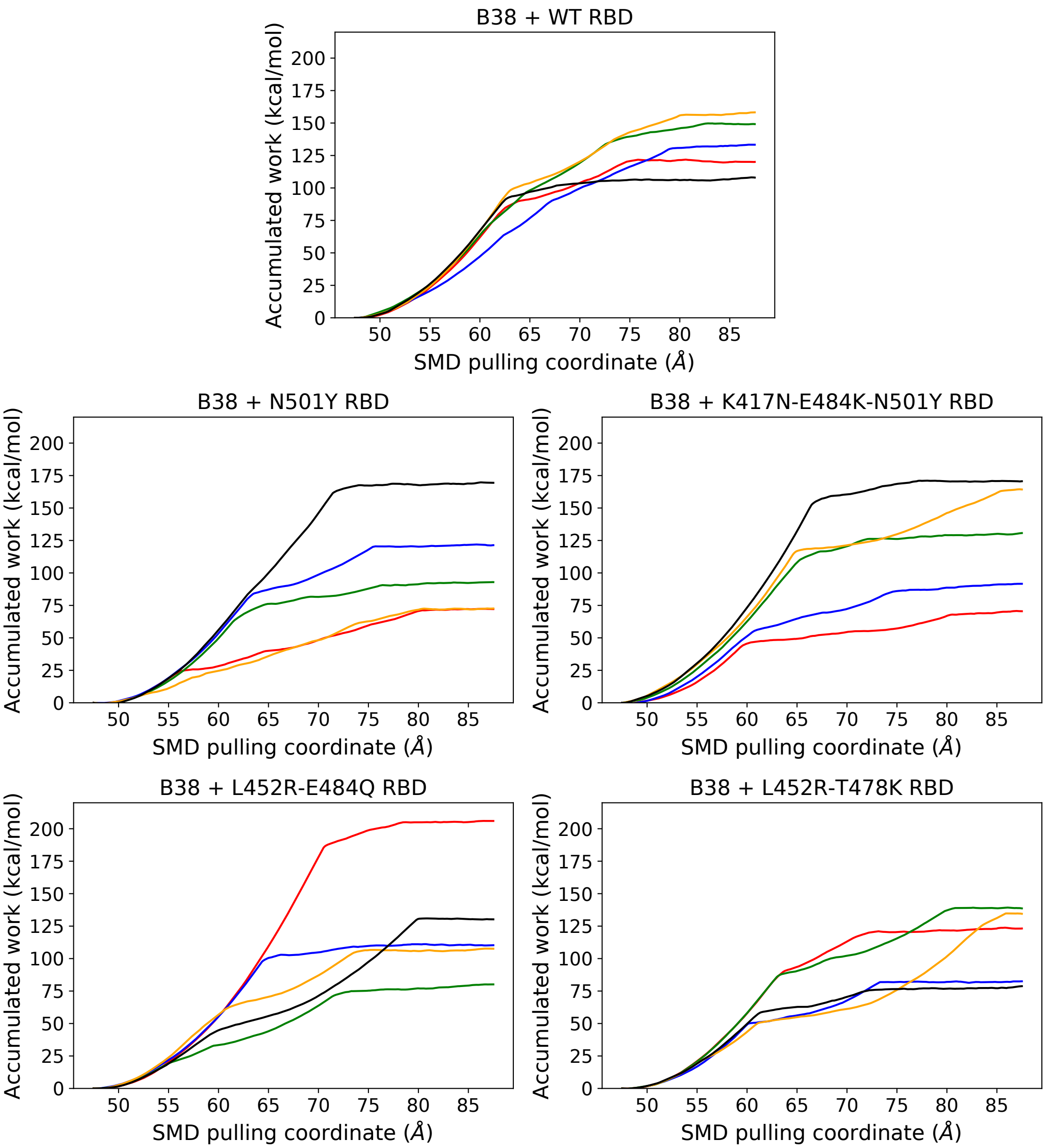

Figure S18: The work required to pull the B38 antibody along the center of mass distance between the RBD and antibody in the SMD simulation. The starting structures for each SMD simulation is sampled from long unbiased simulation after 300, 350, 400, 450 and 500 ns. The color scheme for these five SMD trajectories are as follows: red: 300 ns, blue: 350 ns, green: 400 ns, orange: 450 ns, and black: 500 ns.

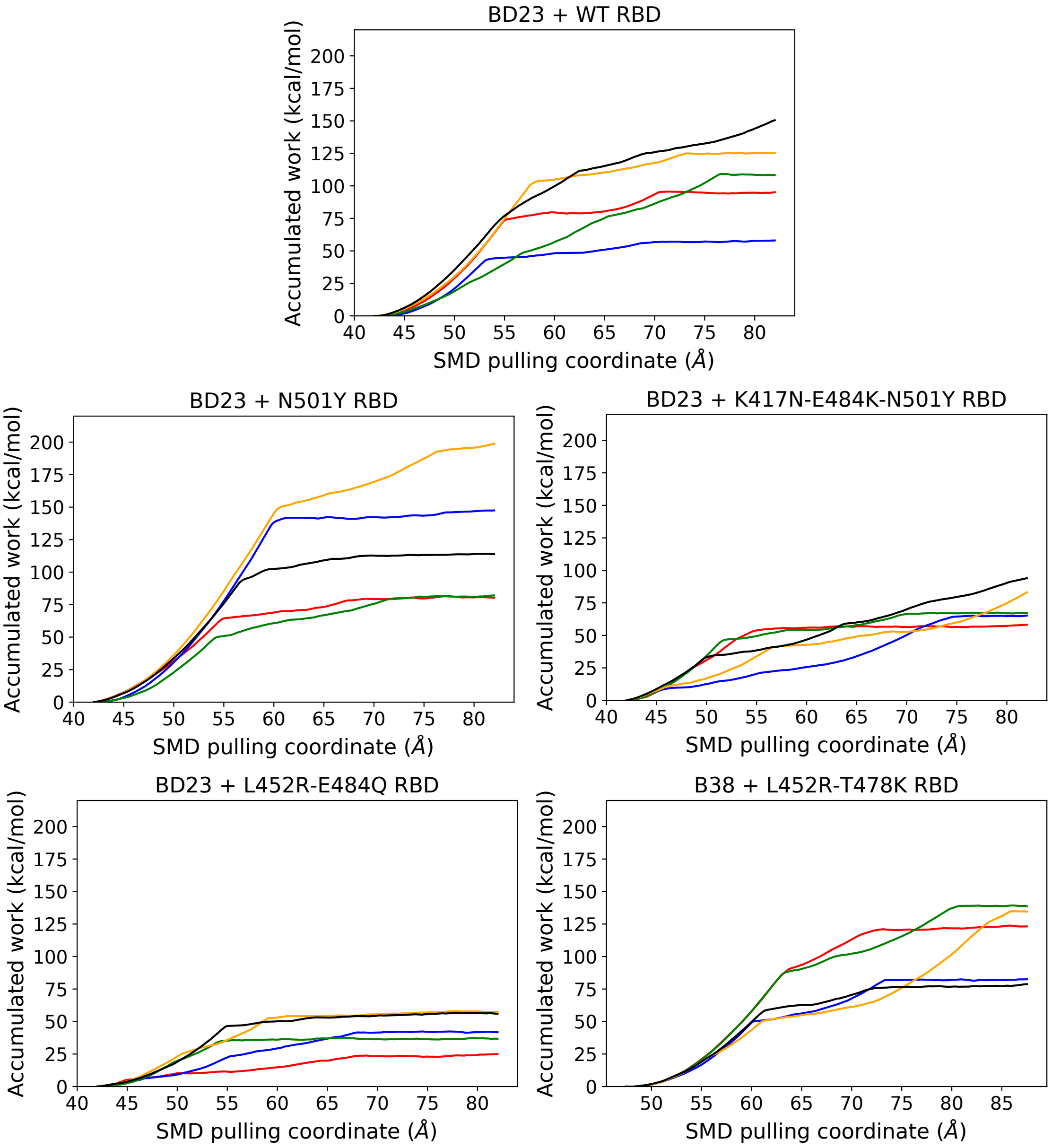

Figure S19: Identical to Figure S18, except for the antibody BD23 Fab.

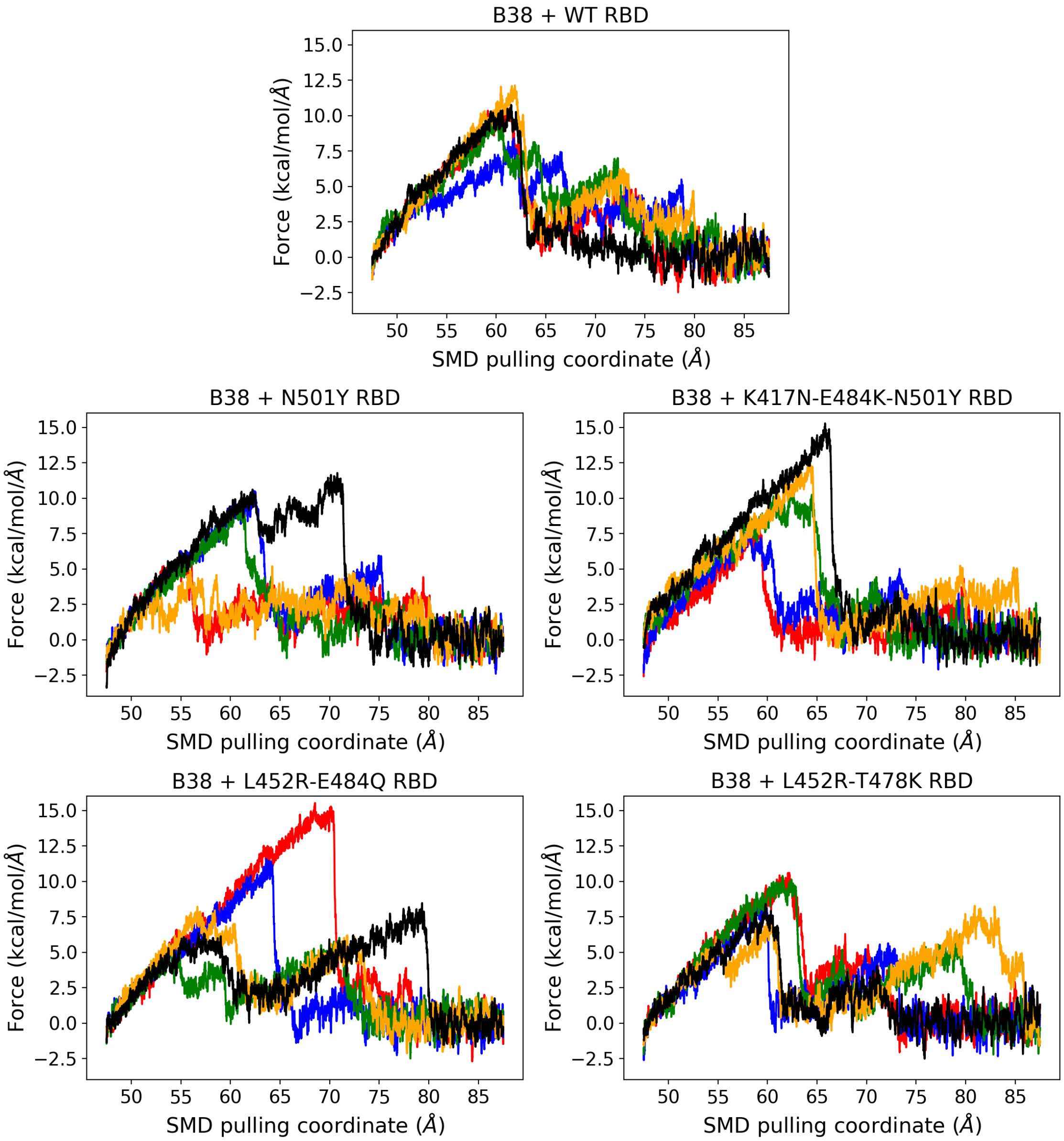

Figure S20: The pulling force required to move the B38 antibody away from the WT and the mutant RBDs along the center of mass distance between RBD and Ab. The color scheme is same as Fig. S18

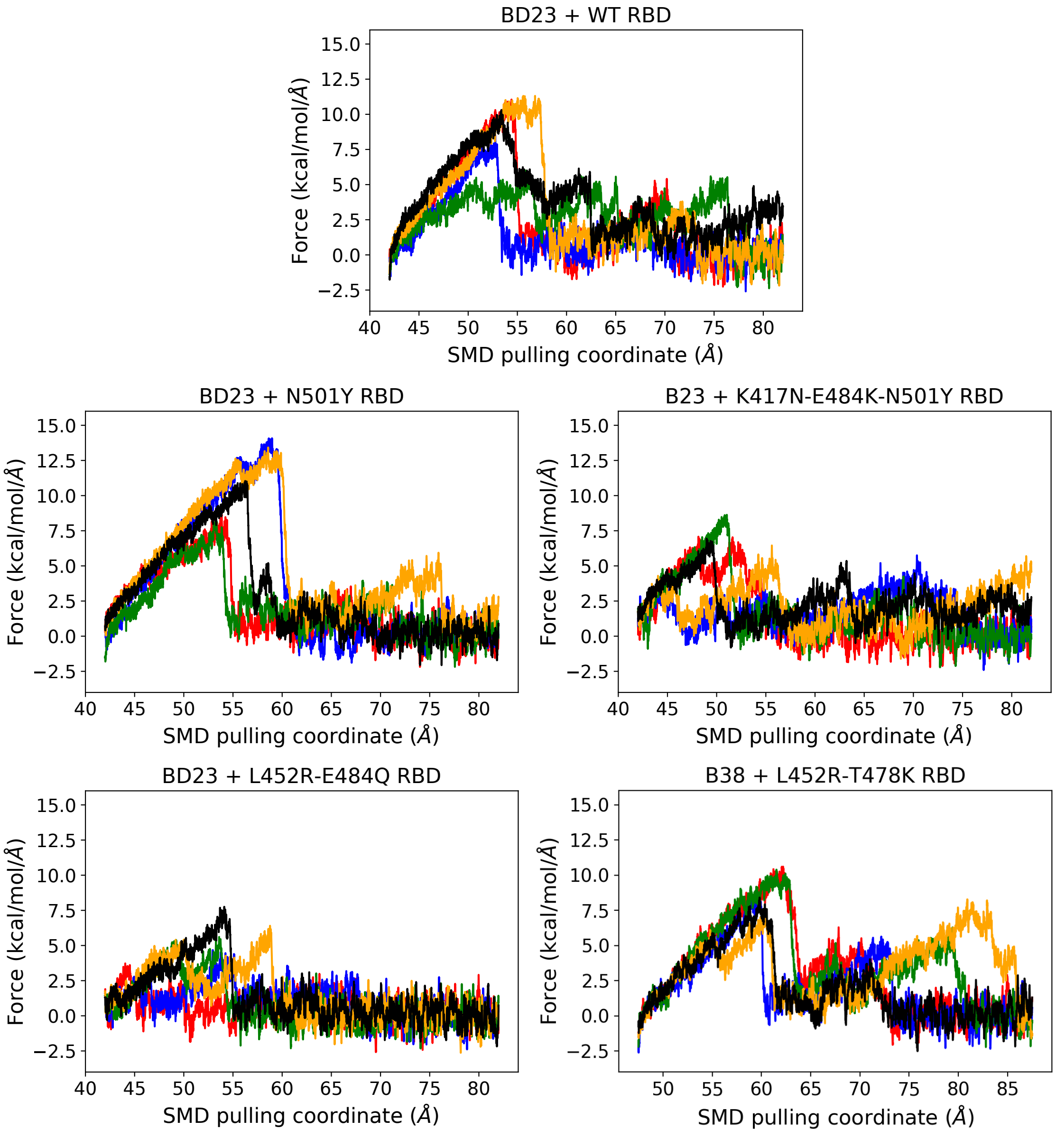

Figure S21: Identical to Figure S20, except for the antibody BD23 Fab.

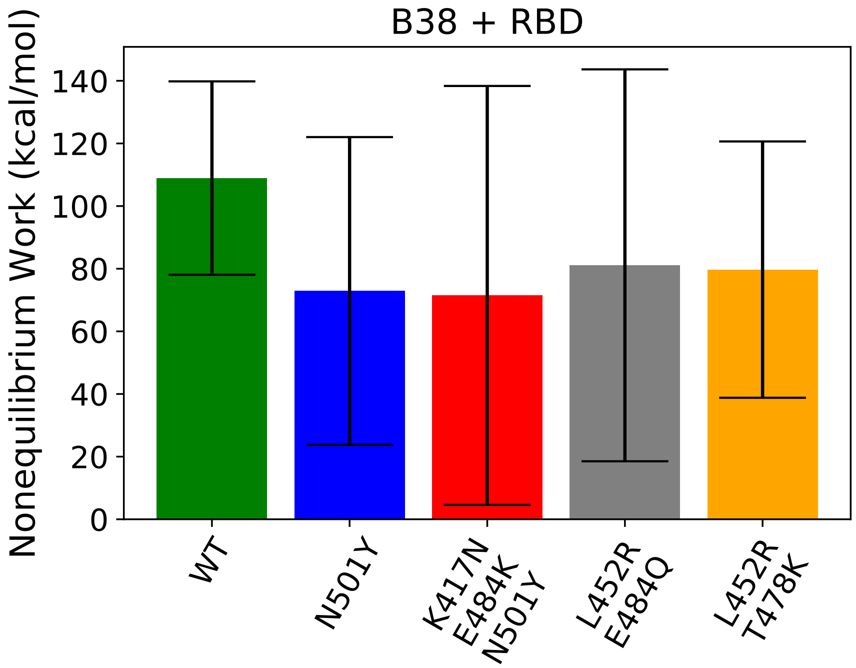

Figure S22: Average non-equilibrium work for the dissociation of the B38 antibody from the RBD, along with error bars (root mean squared errors).

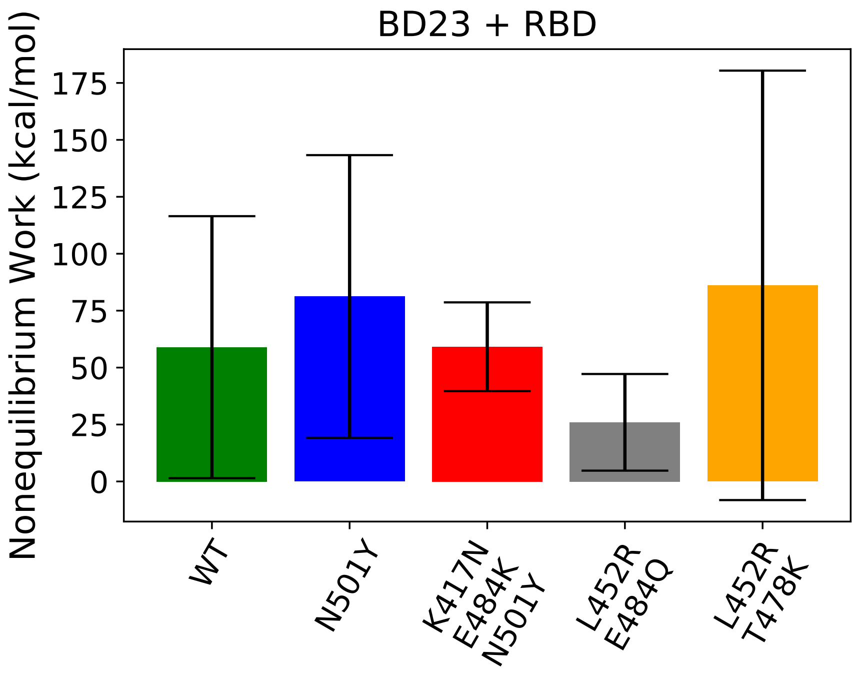

Figure S23: Average non-equilibrium work for the dissociation of the BD23 antibody from the RBD, along with error bars (root mean squared errors).

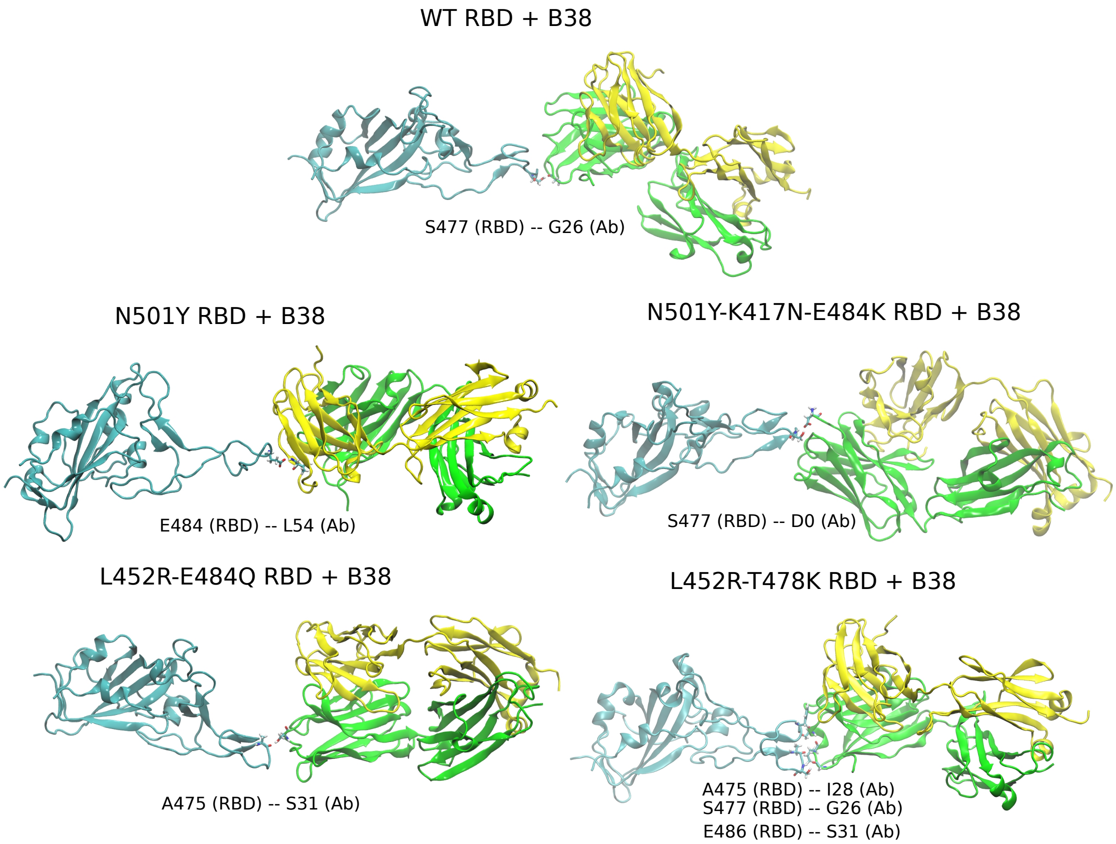

Figure S24: The intermediate structures of the B38 dissociation from the RBD of different variants, sampled from the lowest work SMD trajectory for each respective system. The figure depicts the last intact hydrogen bond between RBD and antibody during the dissociation process.

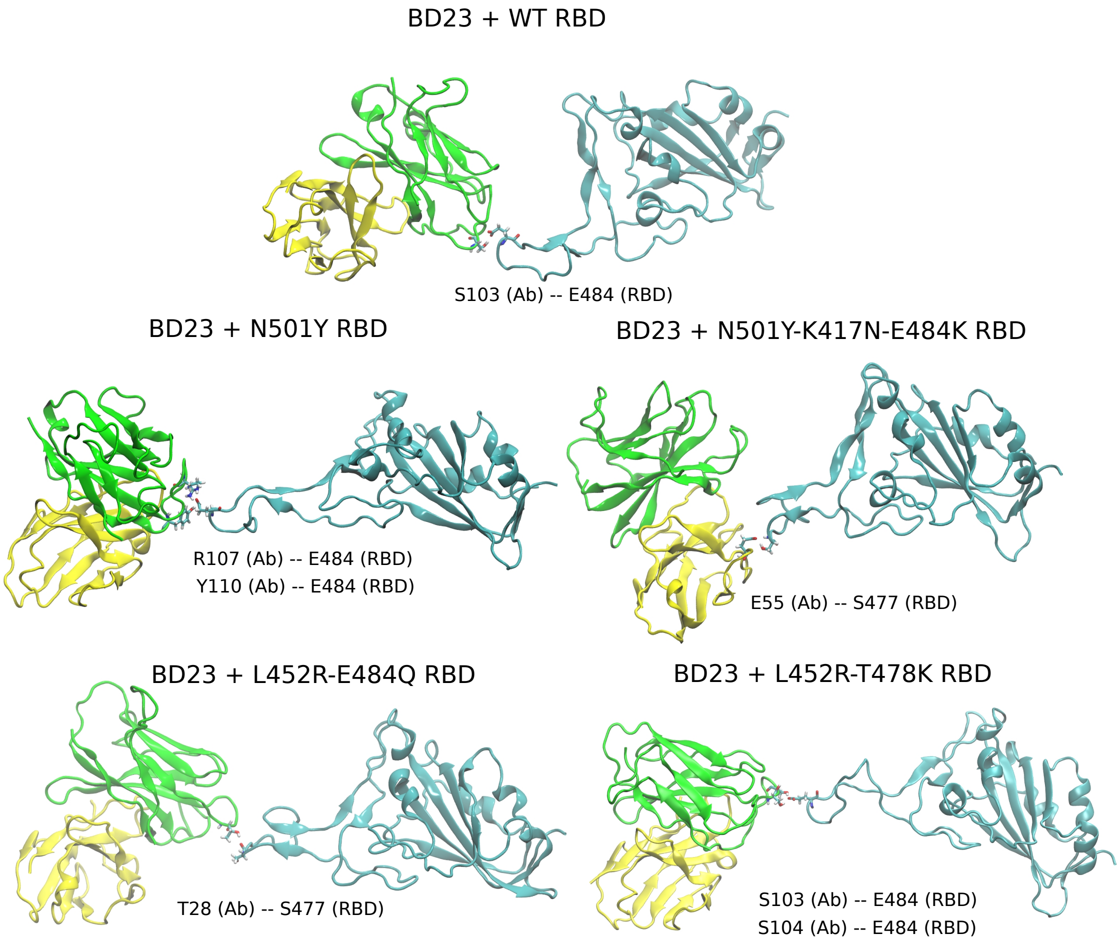

Figure S25: Identical to Figure S24, except for the antibody BD23 Fab.

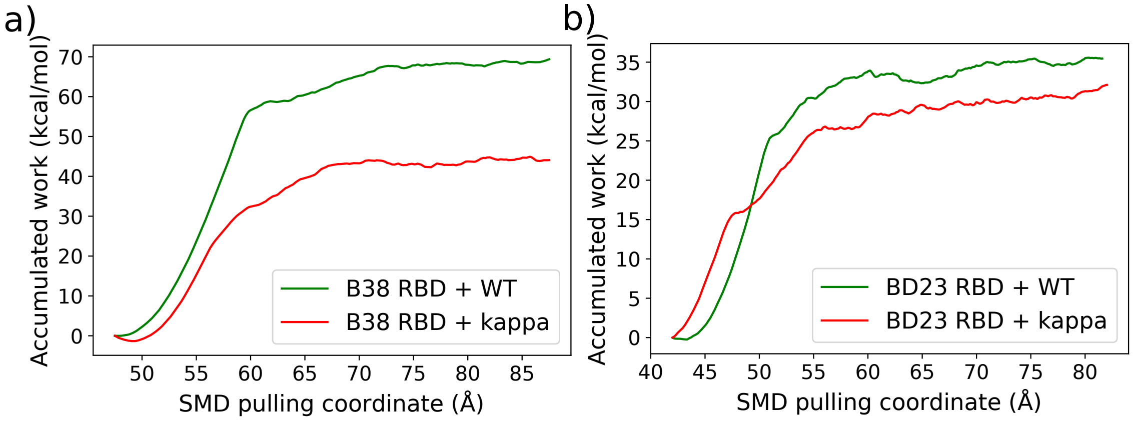

Figure S26: The work required to pull the (a) B38 antibody or the (b) BD23 antibody, along the center of mass distance between the RBD and antibody using *explicit solvent* SMD simulation.

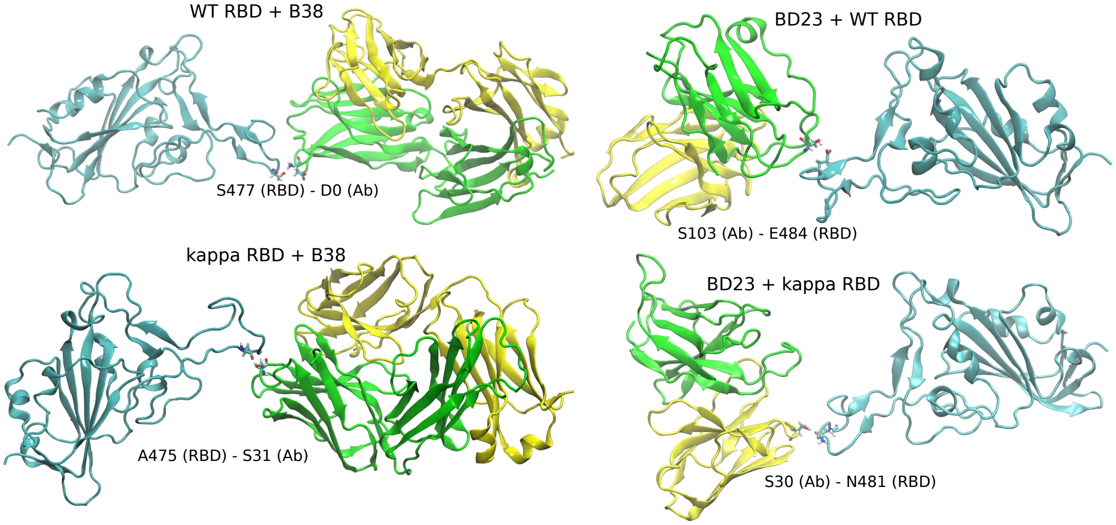

Figure S27: The intermediate structures for the dissociation of the B38 and the BD23 antibodies from the RBD of the WT and the *kappa* variants of the SARS-CoV-2 spike protein, in explicit solvent. The water and ions are not shown for clarity.
